# Hemodynamic effects of early endotoxemia on pulse pressure variation during experimental hemorrhagic shock

**DOI:** 10.1186/cc9468

**Published:** 2011-03-11

**Authors:** J Noel-Morgan, DT Fantoni, DA Otsuki, JO Auler

**Affiliations:** 1 Faculdade de Medicina da Universidade de São Paulo, Brazil

## Introduction

Although pulse pressure variation (PPV) is essentially proposed as a predictor of fluid responsiveness [1], it has also been appointed as an early detector of hypovolemia [2]. Still, caution has been recommended for its employment in certain conditions, as during pulmonary hypertension (PH) [2,3]. Endotoxin-induced PH produces biphasic increase in mean pulmonary artery pressure (MPAP) in several animal models, in which the early phase is acute and transient [4]. The objective of this study was to analyze the early hemodynamic effects of endotoxemia on PPV, during severe hypovolemic shock.

## Methods

Fifty-one anesthetized, mechanically ventilated pigs were randomly allocated to four groups: control (*n *= 8), intravenous endotoxin (*n *= 8), hemorrhagic shock (50% blood volume in 20 minutes; HEM, *n *= 8) or hemorrhagic shock with endotoxin (H+L, *n *= 27). Hemodynamic parameters, measured by pulmonary artery and femoral arterial catheters, were assessed at baseline (TB) and at 20 (T20), 40 (T40), 60 (T60) and 80 (T80) minutes. Groups and times were compared with two-way ANOVA followed by Tukey test (*P *< 0.05).

## Results

At T20, the systolic volume index in groups HEM and H+L dropped significantly (*P *< 0.001), with no difference between groups. MPAP was significantly higher in group H+L than in HEM at T20 (*P *< 0.001), T40 (*P *< 0.001), T60 (*P *= 0.009) and T80 (*P *= 0.013). Within group H+L, MPAP was significantly above TB in all timepoints, but was highest at T20 and T40 (36 ± 13 and 34 ± 7 mmHg, respectively), decreasing significantly at T60 and T80 (to 26 ± 5 mmHg). PPV increased significantly in groups HEM and H+L (both *P *< 0.001) from T20 to T80. There was, however, a statistical difference between HEM and H+L at T20 (27 ± 13% vs. 20 ± 8%, respectively; *P *= 0.044) and T40 (27 ± 7% vs. 18 ± 7%; *P *= 0.006), which disappeared at T60, when PPV in group H+L increased further.

## Conclusions

Even though PPV was affected by the magnitude of MPAP during the peak hemodynamic effects of early endotoxemia, its ability to detect acute decreases in preload was not entirely compromised, in the conditions of the present study. Additional research should help determine possible associated factors that interfere with PPV in related conditions.
